# Subject-independent EEG classification based on a hybrid neural network

**DOI:** 10.3389/fnins.2023.1124089

**Published:** 2023-06-02

**Authors:** Hao Zhang, Hongfei Ji, Jian Yu, Jie Li, Lingjing Jin, Lingyu Liu, Zhongfei Bai, Chen Ye

**Affiliations:** ^1^Translational Research Center, Shanghai Yangzhi Rehabilitation Hospital (Shanghai Sunshine Rehabilitation Center), School of Electronic and Information Engineering, Tongji University, Shanghai, China; ^2^Department of Neurology and Neurological Rehabilitation, Shanghai Disabled Person’s Federation Key Laboratory of Intelligent Rehabilitation Assistive Devices and Technologies, Yangzhi Rehabilitation Hospital (Shanghai Sunshine Rehabilitation Center), School of Medicine, Tongji University, Shanghai, China; ^3^Neurotoxin Research Center of Key Laboratory of Spine and Spinal Cord Injury Repair and Regeneration of Ministry of Education, Neurological Department of Tongji Hospital, School of Medicine, Tongji University, Shanghai, China

**Keywords:** electroencephalograph (EEG), motor imagery (MI), subject-independent, brain-computer interface, generative adversarial networks (GAN)

## Abstract

A brain-computer interface (BCI) based on the electroencephalograph (EEG) signal is a novel technology that provides a direct pathway between human brain and outside world. For a traditional subject-dependent BCI system, a calibration procedure is required to collect sufficient data to build a subject-specific adaptation model, which can be a huge challenge for stroke patients. In contrast, subject-independent BCI which can shorten or even eliminate the pre-calibration is more time-saving and meets the requirements of new users for quick access to the BCI. In this paper, we design a novel fusion neural network EEG classification framework that uses a specially designed generative adversarial network (GAN), called a filter bank GAN (FBGAN), to acquire high-quality EEG data for augmentation and a proposed discriminative feature network for motor imagery (MI) task recognition. Specifically, multiple sub-bands of MI EEG are first filtered using a filter bank approach, then sparse common spatial pattern (CSP) features are extracted from multiple bands of filtered EEG data, which constrains the GAN to maintain more spatial features of the EEG signal, and finally we design a convolutional recurrent network classification method with discriminative features (CRNN-DF) to recognize MI tasks based on the idea of feature enhancement. The hybrid neural network proposed in this study achieves an average classification accuracy of 72.74 ± 10.44% (mean ± std) in four-class tasks of BCI IV-2a, which is 4.77% higher than the state-of-the-art subject-independent classification method. A promising approach is provided to facilitate the practical application of BCI.

## 1. Introduction

Brain-computer interface (BCI) provides an advanced approach that enables users to communicate with external devices (Pfurtscheller and Neuper, 2001). BCIs have shown great potential in many clinical applications, such as controlling assistive robots (Liu et al., 2019) or wheelchairs (Zhang et al., 2016) to help move, drink, and provide stroke rehabilitation, or communicating with others by spelling (Neuper et al., 2006). A variety of physiological information is employed in the BCI systems, and growing attention has been paid to the analysis of electroencephalography (EEG) signals, especially motor imagery (MI), which is one of the most popular paradigms (Pfurtscheller and Neuper, 2001; LaFleur et al., 2013; Kim et al., 2015; Hamedi et al., 2016) due to its portable and cost-effective acquisition system as well as zero clinical risks.

For the past few years, there have been outstanding outcomes in EEG-based classification of MI tasks (Herman et al., 2008; Suk and Lee, 2013; Tabar and Halici, 2017; Jiang et al., 2020). However, most of the current advanced works concentrate on subject-dependent scenario, where data from the same group of subjects is used for training and testing (Zhang et al., 2019). Under the circumstances, a calibration procedure is indispensable to collect sufficient data to build a subject-specific adaptation model employed by a new user, which is time-consuming and labor-intensive. And collecting sufficient data for adaptation can be a huge challenge for stroke patients. Hence, it is imperative to explore the subject-independent scenario for the scalability and usability of BCIs. Due to the high variability and instability of the EEG signals, data from diverse subjects are different, or even at different times on the same session for the same subject. This poses a significant challenge for subject-independent researches.

Most of the conventional MI-based BCIs are exploited from subject-specific approaches, which demand calibration time. One of the most widespread approaches in MI-based BCIs, testified by 2003 BCI competition (Blanchard and Blankertz, 2004), is known as common spatial patterns (CSPs) (Ramoser et al., 2000), which can maximize the variance of one class and minimize the variance of the other for the binary classes. Based on CSP methods, many advanced algorithms have been developed. For example, Lemm et al. (2005) proposed common spatio-spectral pattern (CSSP), which is developed from the CSP method with embedding time delay to extract robust features. In research (Novi et al., 2007), the sub-band common spatial pattern (SBCSP) is proposed to avoid a time-consuming fine-tuning process by applying the CSP algorithm to different sub-bands decomposing the original EEG signal by using a filter bank. Ang et al. (2008) proposed another multiple sub-band input method that is termed the filter bank common spatial pattern (FBCSP), which applies a characteristic picking algorithm to automatically selected discriminative CSP features of different sub-bands. In order to find the optimal filter bank to obtain the discriminative features, Suk and Lee (2013) proposed the Bayesian spatio-spectral filter optimization (BSSFO) that constructs a data-driven discriminative filter bank and bandwidth picking to optimize spatio-spectral filter within a Bayesian framework. Although the efficiency of CSP algorithms is well known and widely used, CSPs are also considered to be very sensitive to noise and prone to overfitting. Improved regularized CSPs have also been proposed recently. Lotte and Guan (2010) proposed CSP with Tikhonov regularization and weighted Tikhonov regularization and demonstrated its advanced performance by comparing them with various RCSP algorithms. Miao et al. (2019) proposed a novel RCSP method to optimize feature extraction and perform MI-BCI classification using the AdaBoost algorithm. A novel regularized common spatial pattern (RCSP) method was also utilized in Jin et al. (2019) to extract effective features to improve the classification accuracy of the MI task. However, these approaches have focused on constructing a pattern classifier to decode the brain patterns specific to the subjects and a calibration procedure is still required to train the decoder.

In recent years, deep learning techniques have attracted significant attention for their success in computer vision, natural language processing (LeCun et al., 2010; Schmidhuber, 2015; Voulodimos et al., 2018; Nassif et al., 2019). Researchers have proposed a few end-to-end deep learning frameworks for subject-independent EEG classification based on MI. Yang et al. (2018) proposed a framework that combines a long short-term memory network (LSTM) with a convolutional neural network (CNN) to simultaneously learn spatial information and capture temporal dynamics from the raw MI-EEG signals, which was employed in subject-independent MI decoders. To further explore the temporal correlation of an MI-EEG sequence, a recurrent-attention networks combined with CNN is developed to focus on most discriminative features in research (Zhang et al., 2019). In research, Kwon et al. (2020) proposed a framework for spectral-spatial feature representation based on deep CNN, which concatenates and fuses spectral-spatial features of discriminative frequency bands by applying spatial fusion technique, and validated the effectiveness on a self-built large MI database. These proposed methods demonstrate the potential of deep learning frameworks for subject-independent EEG classification, but the improvement in subject-independent EEG classification performance is limited due to shortcomings in discriminative feature extraction or dataset size. Due to the powerful feature learning capabilities of deep learning, separable features can be effectively obtained by deep learning approaches with multi-layer nonlinear information processing (LeCun et al., 2010; Deng and Yu, 2014).

However, the performance of deep learning models depends heavily on the scale of the dataset (Abdar et al., 2021). For target subjects, especially stroke patients, collecting sufficient EEG data for adaptive training is a huge challenge. Many researchers have conducted studies of cross-subject EEG classification problems using EEG expansion data collected from other subjects, which has been effective to some extent; however, due to the non-stationary nature of the EEG signal, there are significant individual differences caused by different physiological characteristics. Therefore, the method of data enhancement via EEG from other subjects is limited. On the other hand, the EEG signal has a low signal-to-noise ratio and is susceptible to interference from noise such as impedance and muscle artifacts. When subjects are inattentive during the experiment, they are easily involved in a large amount of irrelevant information. Hence, acquiring sufficient data for adaptation training and extracting effective discriminative features from the low signal-to-noise ratio EEG signal are two major issues affecting subject-independent classification.

With an emphasis on data generation, generative models offer a potential solution to the problem of data deficiency. In particular, GAN has been very successful in computer vision fields, such as image translation (Zheng et al., 2021; Yang et al., 2022) and video generation (Chen et al., 2020; Liu et al., 2021; Wang et al., 2021), etc., due to its excellent artificial image generation capabilities (Saxena and Cao, 2021). However, since EEG is a multi-channel time series signal and is susceptible to interference, a few studies have reported the utilization of GAN for EEG feature or raw data enhancement. Luo et al. (2020) performed enhancement of the power spectral density and differential entropy of EEG signals using a conditional Wasserstein GAN to aid in emotion recognition. In research (Zhang and Liu, 2018), Zhang et al. employed a conditional deep convolution GAN following a wavelet transform to augment the feature data. In addition to generating EEG features, researchers have also attempted to generate unwashed EEG signals for a wider purpose. Hartmann et al. (2018) proposed an EEG-GAN to produce single-channel EEG signals with very well-examined visuals. Roy et al. (2020) used long short-term memory networks in the generator and discriminator and acquired MI EEG signals which have the same characteristics of dynamic and time-frequency as the raw signals. These studies confirm the potential of GAN in generating MI EEG signals, but few studies have used GAN for subject-independent classification due to the high variability and individual differences in EEG signals.

In this paper, we propose a novel hybrid neural network framework based on data augmentation and feature enhancement for subject-independent EEG classification, which first employs filter bank GAN (FBGAN) for data augmentation and obtains high-quality data by adversarial training of generators and discriminators. Specifically, MI EEG are filtered using a filter bank approach, and then sparse CSP features extracted from the multiple sub-bands of filtered EEG data are used as part of the discriminator to maintain more spatial features. Meanwhile, we propose a convolutional recurrent network with discriminative features (CRNN-DF) based on the idea of feature enhancement to extract distinguishable features from EEG signals with low signal-to-noise ratio to identify MI tasks. Furthermore, we have evaluated and analyzed the proposed hybrid neural network from different perspectives and the results show that it offers a promising approach for the study of cross-subject EEG classification problems and for facilitating the practical application of BCI systems. The major innovations and contributions of this work can be summarized as follows: (1) We applied a filter bank approach to extract sparse CSP features from multiple candidate bands. (2) The extracted sparse features were used as part of a discriminator in the proposed FBGAN to inherit more detailed features from the target subjects. (3) We also developed a CRNN-DF classifier based on the idea of feature enhancement to better distinguish MI tasks using extracted discriminative features. (4) Our hybrid neural network framework improves subject-independent EEG classification performance to a conspicuous level through data augmentation and feature enhancement, which helps improve the usability of the BCI system for new users.

The remainder of this paper is organized as follows: Part 2 discusses the methodology of the study. In Part 3, we describe in detail the experiments and results. Details of the experimental analysis are discussed in Part 4. Finally, Part 5 concludes this article.

## 2. Methodology

**Figure 1:**
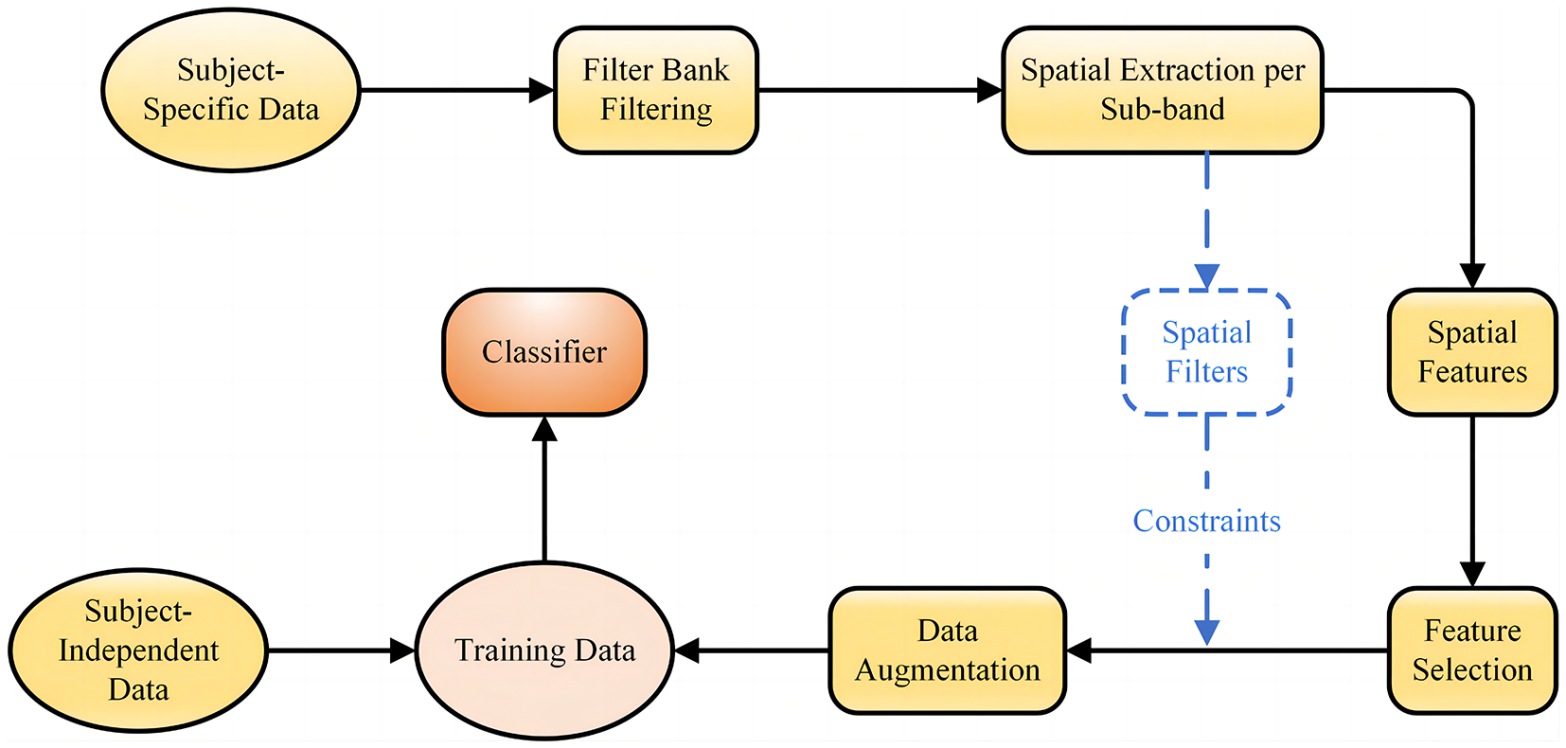
An overview of the hybrid neural network for subject-independent EEG classification.

In practical applications of BCI, good classification results cannot be obtained with subject-independent data only, while calibration with target subject EEG signals requires too much data and it is difficult to extract effective discriminative features from the low signal-to-noise ratio and susceptible to interference EEG signal. In this context, we propose a novel fusion feature network, the general framework of which is shown in Figure 1. First, a filter bank method is used to perform multiple sub-band filtering on the subject-specific EEG data, and each sub-band data is processed to obtain CSP features and spatial filters. Then, lasso regression is used to extract sparse CSP features from the spatial of all frequency bands and acquire the corresponding spatial filters. The sparse spatial features and corresponding spatial filters are then used as constraints for FBGAN for data augmentation. Finally, the augmented data of the target subject is introduced into the subject-independent data for adaptive training, which is applied to the training set of the proposed CRNN-DF.

### 2.1. Data description

The BCI competition IV dataset 2a (Brunner et al., 2008) from Graz University of Technology is applied to verify our approach. The dataset contains EEG signals collected from two sessions of 9 healthy subjects on different days, recording the subjects performing 4 different MI tasks: the movements of left hand, right hand, both feet and tongue, where each session is comprised 6 runs separated by short breaks. One run consists of 48 trials (12 for each of the four classes), yielding a total of 288 trials per session. Two seconds after the start of a trial，a cue corresponding to one of the four classes appeared and stayed on the screen for 1.25 s. The subjects were asked to perform the MI task until the prompt message disappeared from the screen at *t* = 6 s. EEG data were captured by 22 electrodes and sampled at 250 Hz, and then bandpass filtered between 0.5 Hz and 100 Hz. An added 50 Hz notch filter is employed to dampen line noise. In this paper, we represent the samples from each trial as a 2-D matrix $X_{T}^{C}$ , where C is the number of EEG channels and T denotes the sampling points of the EEG data.

### 2.2. Preprocessing

**Figure 2:**
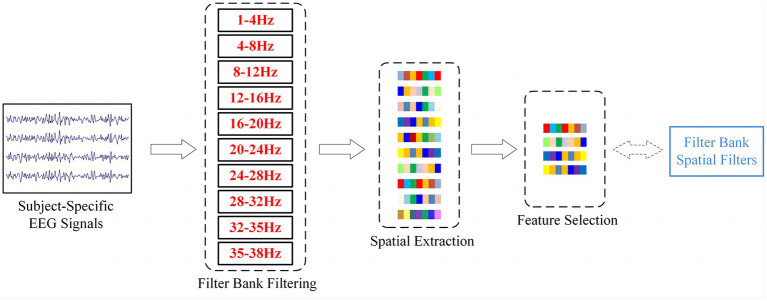
The structural flow of EEG signals processing by the filter bank method. The obtained spatial filters correspond to the sparse CSP features selected by LASSO.

In the raw data, “NaN” was replaced with the average of all sample points. A fifth-order Butterworth bandpass filter from 1 to 38 Hz was applied first to filter out components unrelated to the MI rhythm. The *z*-score standardization was used to reduce the instability and volatility of the EEG signal, which can be expressed as


(1) $$X^{\prime }=\frac{X-\mu }{\sqrt{\sigma^{2}}}$$

where X and $X^{\prime }$ represent the input filtered data and the standardized EEG signal, respectively. μ and $\sigma^{2}$ denote the mean and variance that were calculated by using the training set. Then, the normalized EEG signals were divided into 10 frequency bands (as shown in Figure 2): 1–4 Hz, 4–8 Hz, 8–12 Hz, 12–16 Hz, 16–20 Hz, 20–24 Hz, 24–28 Hz, 28–32 Hz, 32–35 Hz and 35–38 Hz. Finally, a 4-s slice from the start of the cue for each trial was used as a sample.

### 2.3. Feature extraction

CSP is a feature extraction method that is widely used in MI’s BCI and has achieved great success in binary classification problems. It does this by optimizing a set of spatial filters to maximize the variance of one class and minimize that of the other. Since we are faced with a multi-classification task, we employ a modified one-versus-rest (OVR) strategy to overcome the drawbacks of traditional spatial filters. OVR refers to transforming multiple classification problems into multiple binary problems, consisting of one class and the remaining classes. We divide samples of the entire task into 10 sub-bands and compute a sample covariance matrix for each of the four bifurcations in each band. The average spatial covariance matrix can be calculated as


(2) $$R_{c}=\frac{1}{N_{c}}\overset{N_{c}}{\underset{i=1}{\sum }}\frac{X_{i,c}X_{i,c}^{T}}{tr\left(X_{i,c}X_{i,c}^{T}\right)}$$

where $R_{c}$ denotes the mean spatial covariance matrix of class c , $N_{c}$ is the number of trials of class c , $X_{i,c}$ is the i -th trial in class c, and tr() is used to compute the trace of a matrix.

According to Ramoser et al. (2000), we can compute the eigenvector w corresponding to the eigenvalue λ by solving the generalized eigenvalue problem $R_{c}w=\lambda R_{\bar{c}}w$ , where $R_{\bar{c}}$ is the average spatial covariance matrix of the other class. Then, we get a spatial filter for the binary categories in each sub-band. Since there are four classes for the whole task, four sub-filters are obtained for each sub-band. In order to reduce the computational complexity, we remain the four columns corresponding to the four largest eigenvalues in each sub-filter. Thus, there are a total of 4 sub-filters × 4 eigenvectors.


(3) $$W_{csp}^{fr}=\left[w_{1},w_{2},\ldots ,w_{4m}\right]$$

where $W_{csp}^{fr}$ represents the spatial filter obtained from the sub-band fr , and m is the number of eigenvectors retained by the sub-filter in each band. The final spatial filter is then obtained by stacking the sub-filters in each band, with a total of 10 sub-bands × 16 eigenvectors.

### 2.4. Feature selection

By applying CSP to the filtered signal in each sub-band according to the OVR strategy, we can derive the following feature set


(4) $$F=\left[\begin{array}{ccc} f_{1,1} & \cdots & f_{1,D}\\ \vdots & \ddots & \vdots \\ f_{N,1} & \cdots & f_{N,D} \end{array}\right]$$

where $f_{i,j}$ denotes the j -th feature extracted from the filtered EEG signals for the i -th trial, and D=4m×10 is the dimensionality of the feature set. The least absolute shrinkage and selection operator (LASSO) is a penalized least squares method that imposes an L1 penalty on the regression coefficients (Tibshirani, 1996; Zou and Hastie, 2005), which can not only accurately select important variables, but also have the stability of feature selection. LASSO estimation can be formulated as


(5) $$\underset{\beta ,\beta_{0}}{\arg\min}\left(\frac{1}{2N}\overset{N}{\underset{i=1}{\sum }}{\left(y_{i}-\beta_{0}-f_{i}^{T}\beta \right)}^{2}+\lambda \overset{D}{\underset{j=1}{\sum }}\left|\beta_{j}\right|\right)$$

where $y_{i}$ denotes the class label of the i -th trial, $f_{i}$ is the D-dimensional feature vector of the i -th trial, λ is a positive regularization parameter, β is a D-dimensional regression parameter and is a vector, and $\beta_{0}$ is a scalar. The features corresponding to a coefficient of 0 in the LASSO are automatically discarded. Thus, the most important features are selected from multiple frequency bands. We save the spatial filter $W_{csp}$ corresponding to the most important features (as shown in Figure 2), which can be used as


(6) $$Z=W_{csp}^{T}X^{\prime }$$

where Z is the sample processed by the sparse spatial filter $W_{csp}$ .

### 2.5. FBGAN

**Figure 3:**
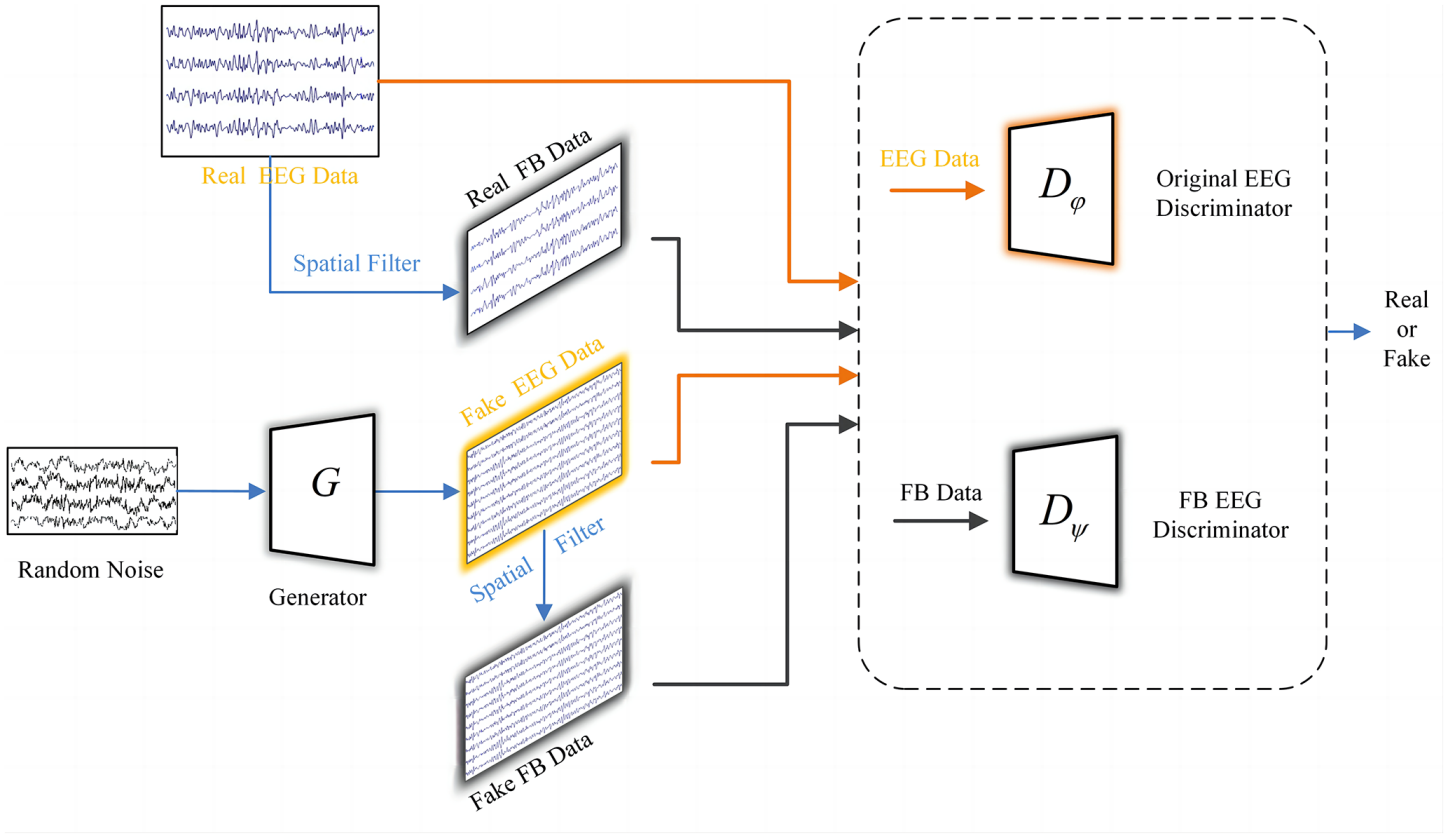
The framework of FBGAN, including a generator and two discriminator modules. Discriminator $D_{\varphi }$ distinguishes between real EEG and generated EEG, and discriminator $D_{\psi }$ is used to distinguish whether the filter bank (FB) data filtered by the sparse spatial filters is real or fake.

In order to inherit more detailed features from the target subject’s EEG signals and prepare sufficient data for adaptive training, we propose FBGAN in the hybrid neural network framework. To the best of our knowledge, it is the first time that the idea of FBCSP has been incorporated into a GAN. Specifically, the MI EEG signals are first filtered in multiple sub-bands, then sparse CSP features are extracted from multiple bands of filtered EEG data, which are used to constrain the GAN to maintain more spatial features of the EEG signal. The architecture of FBGAN is shown in Figure 3. Distinct from the conventional GAN, it includes a generator and two discriminators, and a dedicated discriminator $D_{\psi }$ is innovatively introduced to distinguish the sparse CSP features extracted from the real EEG data and fake EEG data.

GAN consists of a generator ( G ), which learns from random noise to generate artificial data, and a discriminator ( D ), which is used to distinguish artificial data from real data. This can be regarded as a game between G and D . When the game reaches equilibrium, G generates artificial data with a similar distribution to the real data (Goodfellow et al., 2014).

In our framework, the generator is used to generate fake EEG signals with similar distribution to the real EEG. A randomly initialized normally distributed noise (1×1600) to the generator, whose detailed network structure is shown in Table 1, with a fully connected layer FC followed by 5 transposed convolutional layers (ConvTrans). Batch normalization was used to normalize the first four ConvTrans layers. The activation function is LeakyReLU.

**Table 1:** The detailed network structure of the generator $G_{\theta }$ .

Layers	Input	Output	Kernel	Stride	Normalization
FC	1,600	256,000	–	–	–
ConvTrans1	128	128	(3, 15)	(1, 3)	BatchNorm
ConvTrans2	128	128	(3, 15)	(1, 3)	BatchNorm
ConvTrans3	128	64	(3, 5)	(1, 2)	BatchNorm
ConvTrans4	64	32	(4, 5)	(2, 1)	BatchNorm
ConvTrans5	32	1	(1, 2)	(1, 1)	–

Inspired by the study (Song et al., 2021), the discriminator part was specially designed in order to make the generated data inherit the spatial features of the original EEG. The general approach is to distinguish the original data from the generated fake data by a discriminator $D_{\varphi }$ . In our method, in order to preserve more details of the target subjects, we introduce a sparse spatial filter obtained through the feature selection phase to filter the real data and generated data, as in Equation (6). Then, the obtained real and fake filter bank data (FB data) is fed into another discriminator $D_{\psi }$ . The network structure of the discriminator is shown in Table 2, where Conv denotes the convolutional layer, FC denotes the fully connected layer, and Maxpool is the maximum pooling layer. Since each target subject’s EEG has its own specificity, we use an adaptive approach to extract sparse spatial filters using LASSO, rather than extracting a fixed number of filters. Thus, kernel size Var in the third convolution layer of the $D_{\psi }$ adaptively varies according to the dimensionality of the extracted sparse CSP features.

**Table 2:** The detailed network structure of the discriminator $D_{\varphi }$ and $D_{\psi }$ .

Discriminator	Layers	Input	Output	Kernel	Stride	Activation layer
$D_{\varphi }$	Conv1	1	10	(1, 23)	(1, 1)	LeakyReLU
	Conv2	10	30	(22, 1)	(1, 1)	LeakyReLU
	Conv3	30	30	(1, 17)	(1, 1)	LeakyReLU
	Maxpool	–	–	(1, 6)	(1, 6)	–
	Conv4	30	30	(1, 7)	(1, 7)	LeakyReLU
	Maxpool	–	–	(1, 6)	(1, 6)	–
	FC	750	1	–	–	–
$D_{\psi }$	Conv1	1	10	(1, 23)	(1, 1)	LeakyReLU
	Conv2	10	30	(4, 1)	(4, 1)	LeakyReLU
	Conv3	30	30	( Var , 1)	(1, 1)	LeakyReLU
	Conv4	30	30	(1, 17)	(1, 1)	LeakyReLU
	Maxpool	–	–	(1, 6)	(1, 6)	–
	Conv5	30	30	(1, 7)	(1, 1)	LeakyReLU
	Maxpool	–	–	(1, 6)	(1, 6)	–
	FC	750	1	–	–	–

### 2.6. Classifier

**Figure 4:**
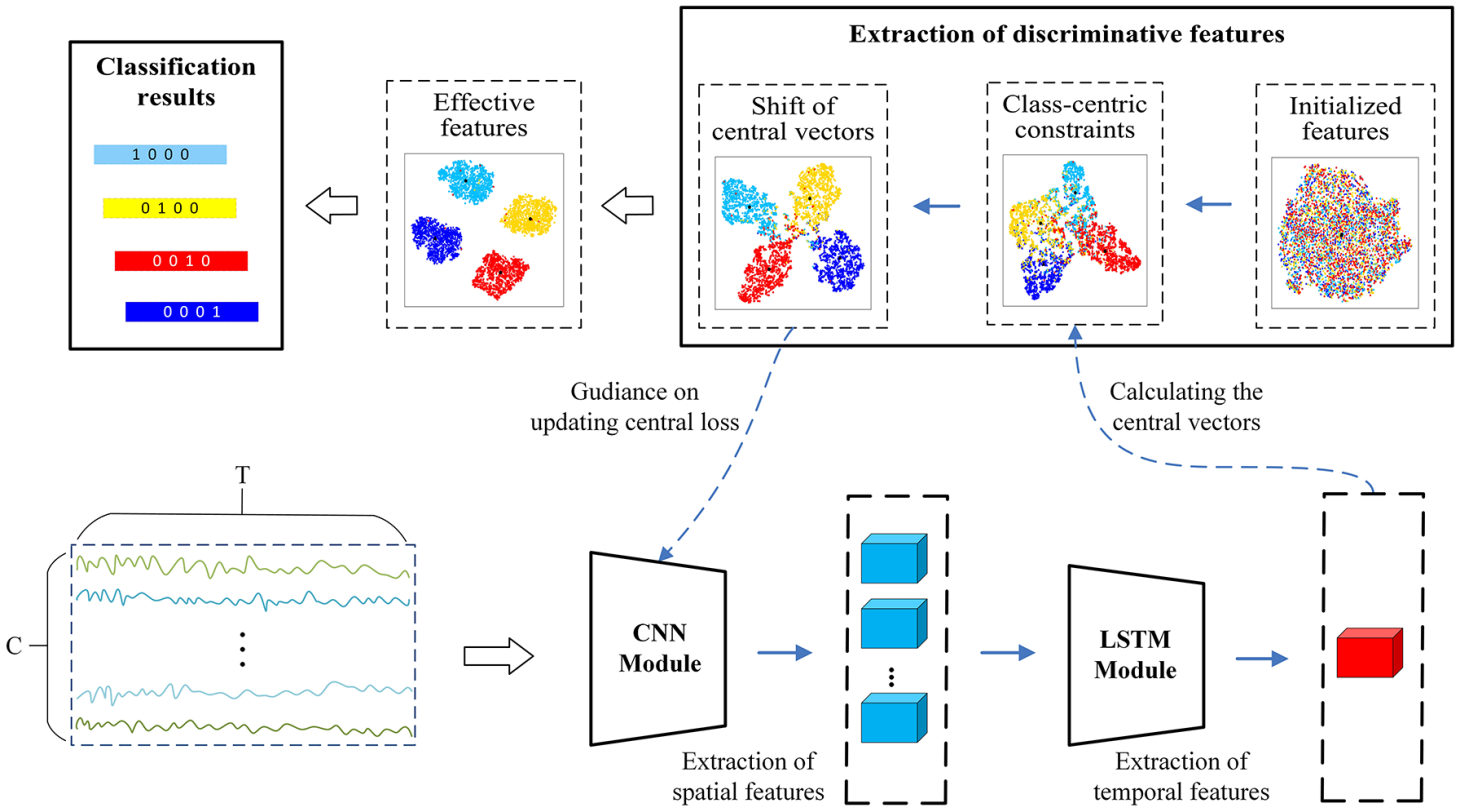
An overview of the CRNN-DF for subject-independent EEG classification.

**Figure 5:**
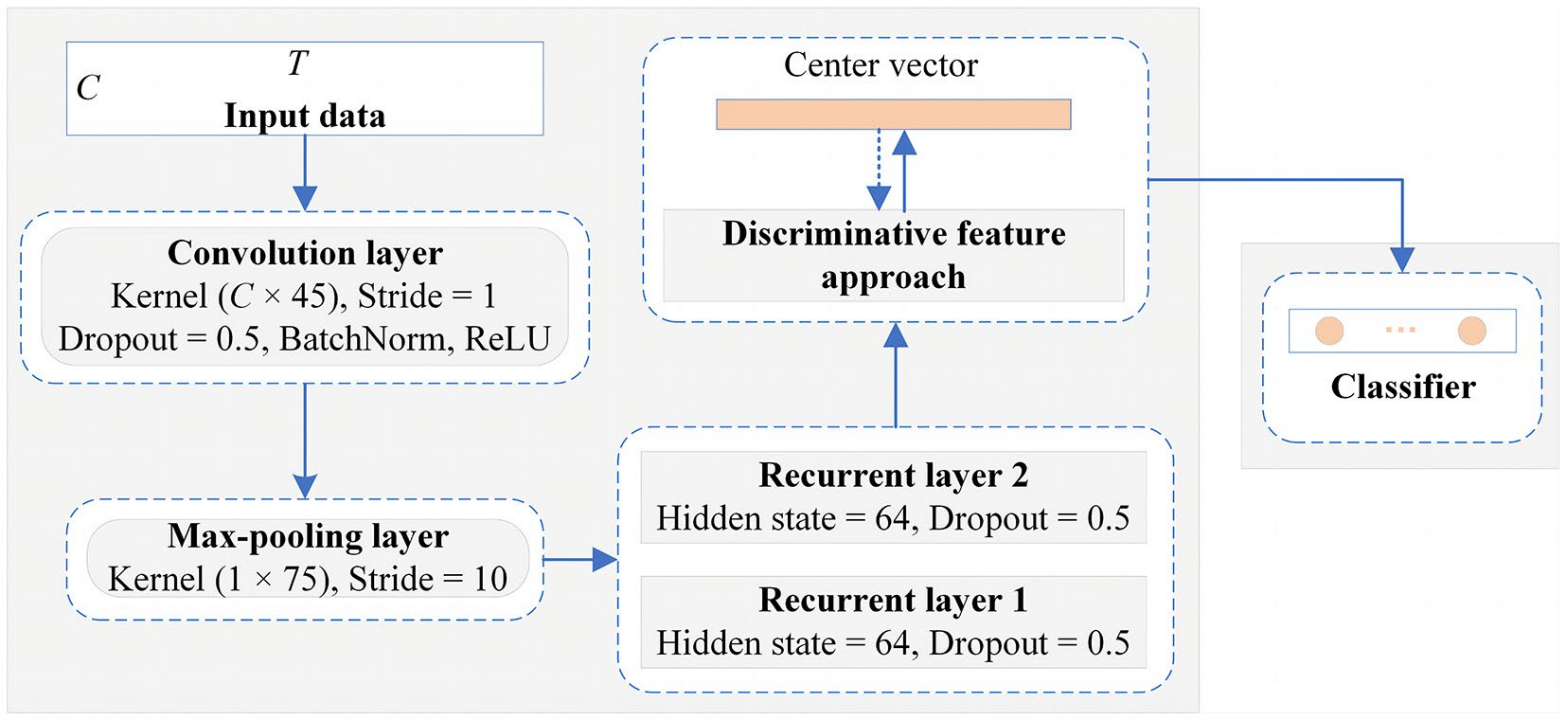
The detailed network architecture of the proposed framework for the classification of subject-independent EEG data.

The EEG samples with the shape C×T are fed into the convolutional module, which conventionally requires a local filter to extract local features from a 2-D matrix. Common local filters for image and video processing are reasonable and successful, such as VGG (Simonyan and Zisserman, 2014), ResNet (He et al., 2016), or AlexNet (Krizhevsky et al., 2017), however, which cannot perform well on raw EEG data. Since EEG signals exhibit diverse characteristics from image and videos, they possess spatial features in one dimension representing the electrode channels and temporal dynamic features in another dimension denoting the time series. Besides, The EEG signals from different electrode channels reflect the functions of different brain regions in the MI task, and there is an intimate relationship between different electrode channels (Ives-Deliperi and Butler, 2018). Therefore, as shown in Figure 4, we apply a convolutional module to extract the spatial features between different electrode channels. The unique convolutional layer in this module has a convolutional kernel size of C×45 and a step size of 1, which can explore the spatial correlation between different electrode channels in the MI tasks. The sample points that are fed into it are encoded as a higher-level representation. Then, a max-pooling layer, which has a kernel size of 1×75 and a step size of 10, is added to reduce the feature dimensionality and the number of parameters. The LSTM module is then employed to explore the temporal dynamics of the features between the different time points. The module consists of two recurrent layers, where the hidden state of each layer is 64. To mitigate overfitting of the classifier during training, the dropout of all network layers is set to 0.5. The detailed structural parameters are shown in Figure 5. Finally, the extraction part of discriminative feature is utilized to improve the discriminativeness of features from different subjects’ EEG data, which is essential for improving the accuracy of the classification of subject-independent EEG signals, which is described in the next subsection.

### 2.7. Extraction of discriminative features

In general, the target function consisting of classification loss is used to guide training of models in classification tasks; however, the features extracted by models trained in this way are usually separable rather than discriminable. CSP maximizes the variance of one category while minimizing the variance of other category to obtain the most discriminative feature vector, which has achieved great success in the two-classification tasks. Inspired by this, we introduce a novel discriminative feature approach (Yang et al., 2021) into our model for subject-independent EEG data classification, which narrows the intra-class diversity and expands the inter-class distance to make the extracted features more discriminative. The brief steps of the method are described as follows.

First, a center vector is computed for the feature vectors of each category in a batch of samples, which can be employed to calculate the central distance loss $L_{cen}$ . In the training process, the intra-class distance is reduced by narrowing the distance between the feature vector of each sample and the corresponding center vector in order to centralize the feature distribution of each class.


(7) $$L_{cen}=\frac{1}{b}\overset{b}{\underset{i=1}{\sum }}∥v_{i}^{k}-cen_{y_{i}}^{k}{∥}_{\;2}$$

Where $v_{i}^{k}$ represents the characteristic vector corresponding to the i−th sample within the k−th iteration, b represents a batch number during training, $y_{i}$ indicates the class tag for the i−th sample, and $cen_{y_{i}}^{k}$ denotes the centroid of class $y_{i}$ within the k−th iteration, which will be initialized with the class center vector of all training samples prior to training, and the initialization process is calculated as follows:


(8) $$cen_{j}^{0}=\frac{\sum_{i=1}^{B}\delta \left(y_{i}=j\right)\cdot v_{i}^{0}}{1+\sum_{i=1}^{B}\delta \left(y_{i}=j\right)}$$

Where $cen_{j}^{0}$ denotes the initialized center vector of the class for the label j , B denotes the number of samples in the entire training set, $v_{i}^{0}$ denotes the initial feature vector of the i−th sample, and $\delta \left(y_{i}=j\right)=\{\begin{array}{c} 0,ify_{i}\neq j\\ 1,ify_{i}=j \end{array}$ is utilized to identity whether the samples in the training set belong to a specific class.

Then, the feature vectors of samples are more discriminative by expanding the distance between the center vectors of different classes. The process of increasing the distance of the class center vectors is to first calculate the center $v_{c}^{k}=\frac{1}{C}\sum_{j=1}^{C}cen_{j}^{k}$ ( C is the number of categories), and then to enlarge the distance between the center vectors and the center, calculated as $cen_{j}^{k+1}=cen_{j}^{k}+\alpha \cdot \frac{\vec{v_{c}^{k}cen_{j}^{k}}}{\left|\vec{v_{c}^{k}cen_{j}^{k}}\right|}$ ( α is the step size of the move).

Finally, the joint supervised training with central distance loss and classification loss is used to guide the optimization of the network parameters of the whole framework. The complete objective loss function is $Loss=-\frac{1}{b}\;\;\sum_{i=1}^{b}y_{i}^{\prime }\log\left(y_{i}\right)+\lambda \cdot L_{cen}$ , where $y_{i}$ and $y_{i}^{\prime }$ denote the true class label and the predicted label corresponding to the i−th sample in a batch, respectively, and λ represents the proportion of central distance loss within the entire loss function.

## 3. Experiments and results

### 3.1. Experiment settings

In Brunner et al. (2008), 288 trials from the first session of the same subject are utilized as the training set and 288 trials from the second session are applied for testing. However, for the cross-subject scene, we apply the leave-one-subject-out (LOSO) approach for subject-independent classification of EEG signals, which employs data from eight subjects for training and those from the remaining one subject for evaluation.

For BCI competition IV dataset 2a, the method randomly shuffles the EEG data of 4,608 trials (8 subjects × 2 sessions × 288 trials) of 8 subjects as the training set, and 576 trials from the remaining 1 subject as the test set to evaluate the classifier performance, and then we introduce generated fake samples to expand the training dataset to validate the proposed hybrid neural network framework validity, in which we take the 22 channels × 1,000 time points of each trial as a sample. Samples from the same subject do not appear in both the training set and test sets at the same time.

**Figure 6:**
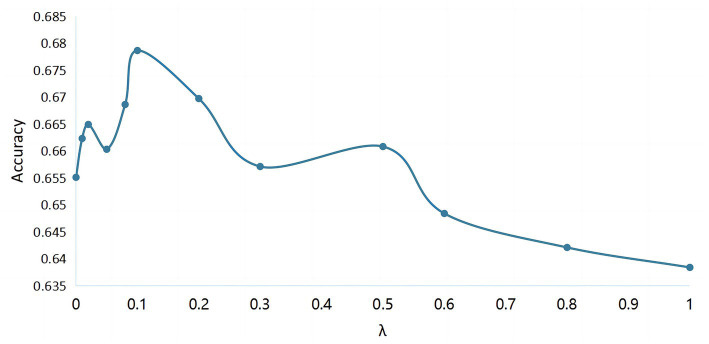
The classification accuracy of cross-subject MI EEG with different values of hyperparameter λ .

The entire neural network structure was implemented by the Tensorflow framework on the Quadro GTX 5000 platform. In FBGAN, an Adam optimizer with a learning rate of 0.0001 was used. The network parameters were updated after a batch size of 5. In classifier, the learning rate and batch size are fixed at 0.0001 and 32, respectively. In addition, the stride of the centric vector transfer for each epoch is 0.02, the central vector is updated every 15 epochs, and the hyperparameter λ of the centric loss in the overall target function is selected experimentally. As shown in Figure 6, when λ is 0, the classifier is equivalent to CRNN without the introduction of discriminative features strategy. And when λ is slightly larger and the value is 0.01, the classification accuracy has a significant improvement. It can be seen in the figure that the recognition rate of the MI EEG tasks is the highest when λ is determined to be 0.1.

### 3.2. Evaluation of the generated data

In order to evaluate the effectiveness of FBGAN for data enhancement, we compared generated signals with original signals of the target subject in terms of time, frequency and spatial domain. As the FBGAN model was parallel for each class of each subject, the training simples and generated simples for subject 9 imagining left-handed movements were averaged separated for visualization.

**Figure 7:**
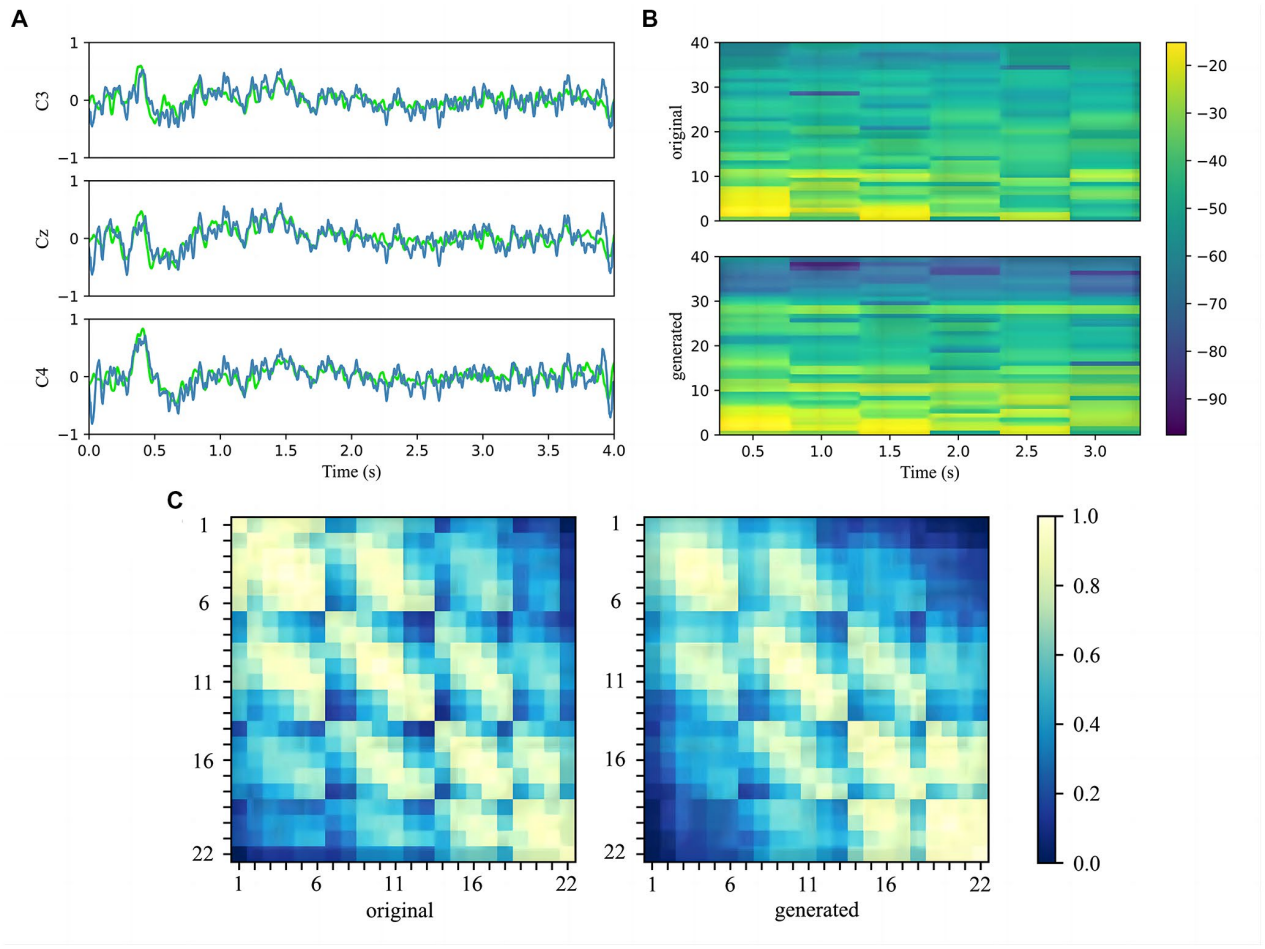
**(A)** Comparison of the C3, Cz, and C4 channels of the original signals and generated signals in the time domain. The original signals are marked by lime and the generated signals are marked by steel blue. **(B)** Comparison of the spectrograms of the original signals and generated signals after the 22 channels data have been averaged. The vertical axis indicates the frequency in Hz, and colorbar is in dB. **(C)** Heat map which compares the covariance matrix of the raw real data and the generated data illustrates the correlation between the electrode channels. Each small block denotes the covariance between the two electrodes.

Firstly, the three main channels C3, Cz, and C4 of the MI region were chosen to compare the original signals and generated signals in the time domain (Pfurtscheller et al., 2006). As shown in Figure 7A, we represent the original data in lime and the generated data in steel blue on the same axis. It can be seen that the generated signals are similar to the real signals in time distribution, and the average and range are quite close.

Secondly, the 22 channels of real and fake samples signals are average to show the power spectrum density by drawing the spectrograms. Figure 7B plots the spectrogram with 1–38 Hz as the pre-processing. It can be noticed that generated data displays higher power where the original data power is higher, especially in the range 1–30 Hz. Since the filtered sub-bands are selected by LASSO during the pre-processing stage, the selected feature band will be paid special attention to the generated model.

Thirdly, the heat map is employed to observe the details of generated data in terms of spatial distribution and to assess quality. The normalized covariance matrix of the original and generated data is plotted in the heat map, as shown in Figure 7C As the covariance matrix reflects the relationship between the data rows, it can be seen from the heat map that the relationship between adjacent electrode channels is well retained, which indicates that generated signals are spatially consistent with original signals.

### 3.3. Classification performance

To verify the effectiveness of the proposed subject-independent classification method CRNN-DF, we conducted a number of experiments on the BCI competition IV 2a dataset and compared them in detail with other advanced methods based on the same dataset, respectively. There are significant individual discrepancies in the EEG signals of different subjects due to their unique physiological structure and psychological state. To adequately validate our method, we trained a model for each subject with LOSO approach to ensure that dataset used for training and testing were from different subjects, respectively.

Table 3 presents subject-independent MI EEG decoding accuracies and their average accuracies from subject A1 to subject A9. In this table, we compared with competitive approaches on the BCI competition IV 2a dataset, including EEGNet (Lawhern et al., 2018), CTCNN (Schirrmeister et al., 2017), AE XGboost (Zhang et al., 2017), FBCSP (Ang et al., 2008), and CRAM (Zhang et al., 2019). From the table, we can observe that our classifier has higher average accuracy than the comparative approaches when tested on all subjects separately. Furthermore, the proposed method achieved the maximum average precision on the 2a dataset.

**Table 3:** Comparison of the subject-independent EEG decoding accuracy (%) with the present advanced classification approaches on the BCI competition IV 2a dataset and A1–A9 denotes nine different subjects.

Comparision method	Test subject (the remaining subjects used as training)									Mean	Std
	A1	A2	A3	A4	A5	A6	A7	A8	A9		
EEGNet	53.76	39.54	54.88	43.02	51.80	48.96	60.70	61.38	47.82	51.32	6.94
CTCNN	55.90	26.04	70.66	45.49	33.16	35.42	40.97	61.29	60.07	47.67	14.20
AE XGboost	32.12	32.34	32.29	32.99	33.85	32.47	39.06	30.90	32.64	33.18	**2.20**
FBCSP	47.92	24.83	39.24	39.93	27.26	31.60	27.08	46.70	36.68	35.69	8.04
CRAM	61.02	42.35	73.11	50.43	50.74	51.48	67.26	69.72	66.85	59.22	10.13
**CRNN-DF**	**65.51**	**45.18**	**78.62**	**53.58**	**55.64**	**56.03**	**71.28**	**75.02**	**70.78**	**63.52**	10.70

### 3.4. Comparison of feature distributions

**Figure 8:**
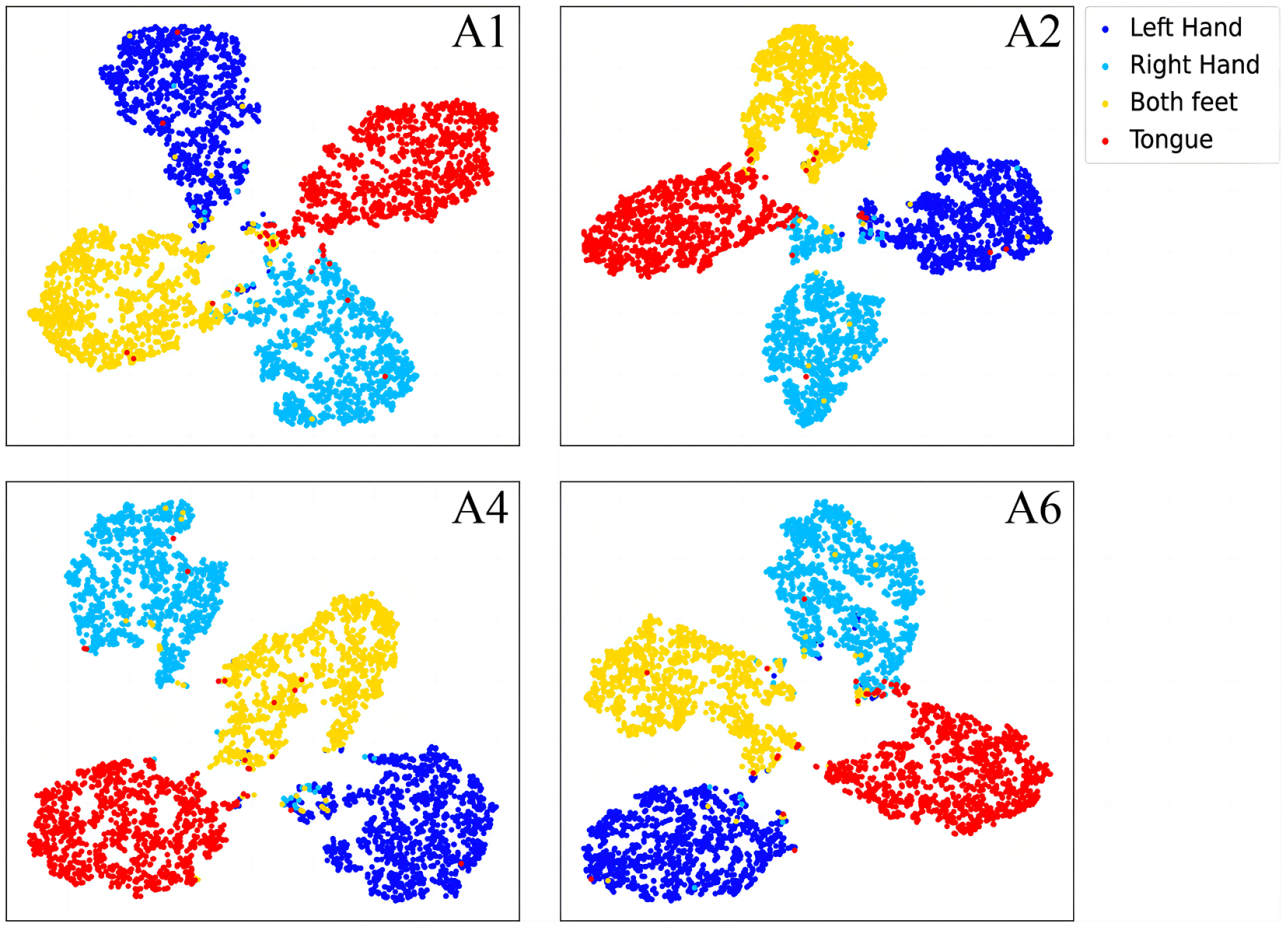
The separative features of typical subjects from the BCI competition IV 2a dataset that are acquired by the proposed convolutional recurrent networks framework, mapped to the two-dimensional plane via TSNE.

**Figure 9:**
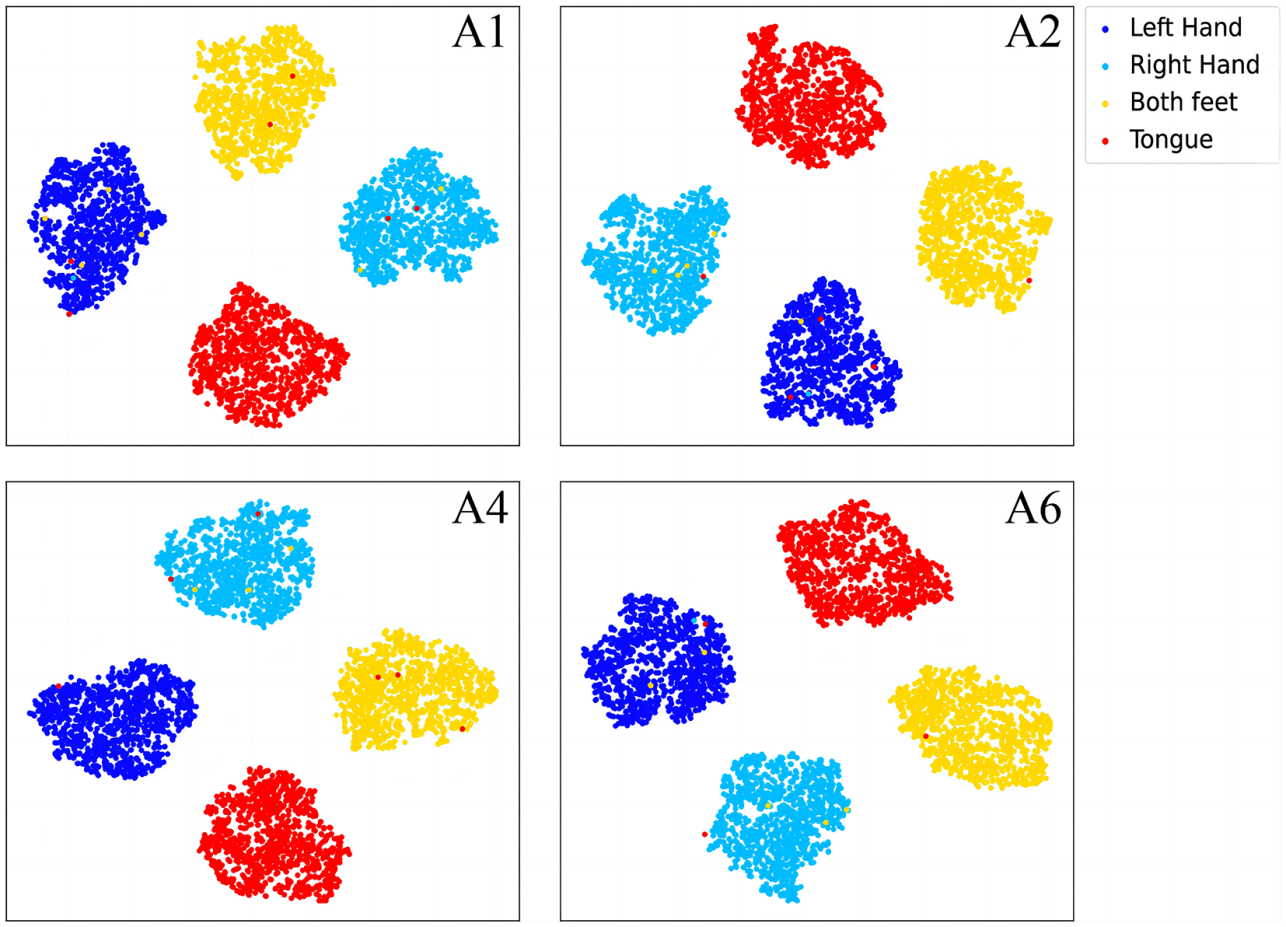
The discriminative features of typical subjects from the BCI competition IV 2a dataset that are acquired by the proposed CRNN-DF, mapped to the two-dimensional plane *via* TSNE.

To further demonstrate the validity of the classification method at the subject-independent EEG feature level, we output feature vectors of typical subjects in 2a dataset. All these vectors are then converted to the two-dimensional plane via TSNE (van der Maaten and Hinton, 2008). As can be seen in Figures 8, 9, the sample features of the subjects are distributed chaotically in the feature space before the processing with the discriminative feature method, and the feature vectors of the different MI tasks are not sufficiently distinguishable. The comparison indicates that our method allows the similar sample features from different subjects to converge to the same area of the characteristic space, and the sample characteristic from diverse categories to become sufficiently discriminative in the feature space, which can help us achieve higher classification accuracy.

### 3.5. Data augmentation for subject-independent classification

After confirming the effectiveness of the designed subject-independent classifier CRNN-DF, we tried to introduce fake data generated for the target subjects in the training set to better help the classifier perceive subject-specific features and separate the four MI categories. The classification results after introducing different numbers of fake data for augmentation are shown in Table 4. Since there are four categories in the MI task, the number of samples in each one is one-fourth of the total number of samples introduced. It can be seen from the table that when only 500 generated fake samples are introduced, the average classification accuracy is greatly improved. As the number of fake samples increases, the accuracy rate has improved to varying degrees. However, for subjects A4 and A8, the accuracy at the introduction of 3,000 samples was lower than that at the introduction of 2000 samples, which may be due to the addition of other irrelevant information along with the target subject features when introducing the generated fake samples. Excessive augmented samples may cause the noise to dispel the effect of the valid information. Therefore, for each target subject, we introduced 3,000 generated fake samples, that is, 750 samples per category in our framework.

**Table 4:** The classification accuracy (%) from subject A1 to subject A9 for different numbers of augmentation samples, where $N_{aug}$ denotes the number of fake samples introduced in the training set and A1–A9 denotes nine different subjects.

$N_{aug}$	A1	A2	A3	A4	A5	A6	A7	A8	A9	Mean	Std
0	65.51	45.18	78.62	53.58	55.64	56.03	71.28	75.02	70.78	63.52	10.70
500	74.01	48.78	80.97	60.51	64.40	57.56	74.45	80.07	76.04	68.53	10.55
1,000	77.32	48.69	81.55	61.64	66.69	58.00	77.10	80.12	78.01	69.90	10.97
2000	77.95	50.37	81.94	63.74	67.35	57.03	79.30	**83.88**	80.24	71.31	11.42
3,000	**79.60**	**54.25**	83.84	60.93	**70.54**	**62.18**	**79.94**	82.01	**81.36**	**72.82**	**10.44**
4,000	79.60	51.94	**85.87**	**65.24**	68.34	61.31	78.39	83.48	81.17	72.82	10.94

**Figure 10:**
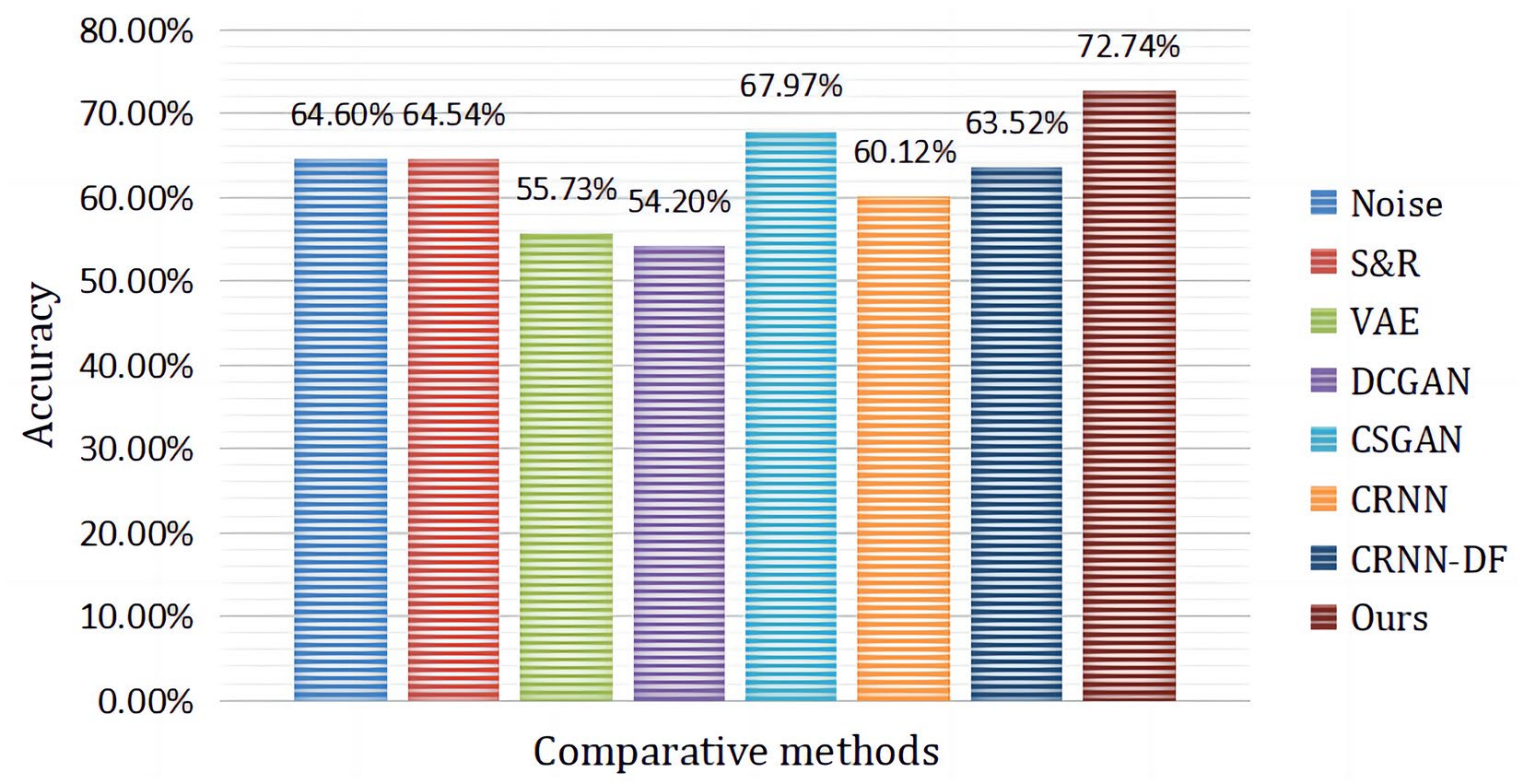
The average classification accuracy of subject A1 to subject A9 compared to advanced augmentation methods, where CRNN-DF is the proposed classifier and no augmented data were used.

Figure 10 presents the comparison of our proposed hybrid neural network framework with the current state-of-the-art subject-independent classification approach. It can be seen from the table that our proposed framework obtains the best classification accuracy. As shown in Table 3, the CRNN-DF classification method designed in this paper obtained satisfactory recognition results with LOSO strategy and without the introduction of augmented data. Then, we further introduced 3,000 fake samples for target subjects, which led to a huge improvement in the results of the four MI classification tasks. It is due to the combination of OVR and CSP in the pre-processing stage of the hybrid framework, which maximized the variance of one class while minimizing the variance of the other, expanding the difference between one and other categories. In addition, the introduction of augmented data from target subjects and the discriminative feature strategy employed in the classification phase played an important role in improving the distinguishability of the different classes.

## 4. Discussion

The brain patterns of different subjects performing the same MI tasks usually have individual differences, and these differences always interfere with the subject-independent MI EEG decoding process, which has long restricted the application of EEG-based BCI. In this study, we proposed a subject-independent hybrid neural network framework to solve the cross-subject classification problem for MI tasks. To overcome the effects of large individual differences, low signal-to-noise ratio, and difficulty in collection in EEG data, we designed FBGAN to generate EEG samples for data augmentation, and designed CRNN-DF to extract effective discriminative features based on the idea of feature augmentation.

In the article, the BCI Competition dataset 2a was employed to evaluate the method performance. As shown in Table 3, the CRNN-DF achieved advanced classification performance with LOSO strategy for each subject and obtained the highest average classification accuracy. This is because the use of the discriminative feature strategy makes the features vectors of the same category sample more compact in the feature space and ones of samples of different classes more dispersive as shown in Figures 8, 9, which improved the resolution of brain patterns across MI tasks and improved generalization to different subject’ brain patterns. To enable the classifier to better perceive subject-specific features, we introduced fake EEG samples of target subjects generated by FBGAN into the training set. As can be seen in Table 4, the average classification accuracy was greatly improved after 500 generated fake samples were introduced. As the number of introduced fake samples increased, the performance of the classifier improved to varying degrees. We also compared FBGAN with some other powerful augmentation methods, such as adding Gaussian Noise, Segmentation and Recombination (S&R) (Fan et al., 2020), Variational Auto-Encoder (VAE) (Bao et al., 2021), Deep Convolutional GAN (DCGAN) (Xu et al., 2022), and Common Spatial GAN (CSGAN) (Song et al., 2021), as shown in Figure 10. The superiority of the proposed method is further demonstrated by the ablation experiments of discriminative feature strategy and FBGAN in hybrid neural networks. Furthermore, as shown in Figure 7, we have analyzed and compared the details of the data generated by FBGAN with the original data in three dimensions: time domain, frequency domain, and spatial domain, which confirms that the generated signals are indeed of sufficient quality.

However, our method still has some limitations. Firstly, as can be seen from Table 3, although the decoding accuracy of our method is the highest on BCI Competition IV dataset 2a, the standard deviation is also relatively large and the stability is not yet good enough. The main reason is that EEG signals vary greatly from subject to subject. Although our method is able to overcome the differences in brain patterns between subjects to some extent, it is not yet well adapted to subjects with large variability. But this problem was alleviated after introducing more generated data from the target subjects due to the enhanced adaptability of the target subjects. Secondly, the introduction of augmented data did significantly improve the classification results for cross-subject MI tasks, but in fact, it can be seen from Table 4 that the quality of the signals generated by FBGAN was not always perfect. For example, the classification results for subject A4 introducing 3,000 samples were worse than those introducing 2000 samples, which is due to the fact that the input noise is high and somewhat random, and the generated signals has certain fluctuations. The balance between the amount of input noise and the diversity of the generated data deserves more research. Thirdly, as the FBGAN model is parallel to each category of each subject, which increases the computational cost.

## 5. Conclusion

In this paper, we present a novel hybrid neural network for subject-independent EEG signal classification. The framework uses a specially designed FBGAN to obtain high-quality EEG data for augmentation. Based on the idea of feature enhancement, the CRNN-DF is designed to recognize MI tasks, which introduces a discriminative feature strategy to expand the inter-class feature differences and narrow the intra-class feature distances. This improves the recognition rate of different subject brain patterns by enhancing the distinguishability between different classes of samples. The experimental results indicated that our method significantly outperforms previous subject-independent methods and can overcome the differences in brain patterns across subjects to some extent. In conclusion, the approach is expected to pave the way for the practical implementation of subject-independent BCI systems, alleviating the mutual interference between different subject brain patterns and improving the accuracy of the EEG decoding process.

## Data availability statement

Publicly available datasets were analyzed in this study. This data can be found here: https://www.bbci.de/competition/iv/.

## Author contributions

HZ carried out experiment and writing. HJ, JY, and JL designed the overall framework. LJ, LL, ZB, and CY carried out methodological guidance and formal analysis. All authors contributed to the article and approved the submitted version.

## Funding

This work was supported by the Shanghai Municipal Science and Technology Major Project (2021SHZDZX0100), the Fundamental Research Funds for the Central Universities, the Science and Technology Innovation Action Plan of the Shanghai Science and Technology Commission (19441908000), and Program of Shanghai Academic Research Leader (20XD1403400).

## Conflict of interest

The authors declare that the research was conducted in the absence of any commercial or financial relationships that could be construed as a potential conflict of interest.

## Publisher’s note

All claims expressed in this article are solely those of the authors and do not necessarily represent those of their affiliated organizations, or those of the publisher, the editors and the reviewers. Any product that may be evaluated in this article, or claim that may be made by its manufacturer, is not guaranteed or endorsed by the publisher.

## References

[ref1] Abdar M. Pourpanah F. Hussain S. Rezazadegan D. Liu L. Ghavamzadeh M. . (2021). A review of uncertainty quantification in deep learning: techniques, applications and challenges. Inf. Fus. 76, 243–297. doi: 10.1016/j.inffus.2021.05.008

[ref2] Ang K.K. Chin Z.Y. Zhang H.H. Guan C.T. (2008). Filter bank common spatial pattern (FBCSP) in brain-computer interface. 2008 IEEE international joint conference on neural networks, Hong Kong 2390–2397.

[ref3] Bao G. Yan B. Tong L. Shu J. Wang L. Yang K. . (2021). Data augmentation for EEG-based emotion recognition using generative adversarial networks. Front. Comput. Neurosci. 15:723843. doi: 10.3389/fncom.2021.723843, PMID: 34955797 PMC8700963

[ref4] Blanchard G. Blankertz B. (2004). BCI competition 2003- data set IIa: spatial patterns of self-controlled brain rhythm modulations. IEEE Trans. Biomed. Eng. 51, 1062–1066. doi: 10.1109/Tbme.2004.826691, PMID: 15188879

[ref5] Brunner C. Leeb R. Müller-Putz G. Schlögl A. Pfurtscheller G. (2008). BCI Competition 2008–Graz data set A. Austria: Institute for Knowledge Discovery (Laboratory of Brain-Computer Interfaces), Graz University of Technology 16, 1–6.

[ref6] Chen Q. Wu Q. Chen J. Wu Q. Y. van den Hengel A. Tan M. K. (2020). Scripted video generation with a bottom-up generative adversarial network. IEEE Trans. Image Process. 29, 7454–7467. doi: 10.1109/TIP.2020.3003227

[ref7] Deng L. Yu D. (2014). Deep learning: methods and applications. Found. Trends Signal Process. 7, 197–387. doi: 10.1561/2000000039

[ref8] Fan J. Sun C. Chen C. Jiang X. Liu X. Zhao X. . (2020). EEG data augmentation: towards class imbalance problem in sleep staging tasks. J. Neural Eng. 17:056017. doi: 10.1088/1741-2552/abb5be, PMID: 33055386

[ref9] Goodfellow I. Pouget-Abadie J. Mirza M. Xu B. Warde-Farley D. Ozair S. . (2014). Generative adversarial nets. Adv. Neural Inf. Process. Syst. 35, 53–65. doi: 10.1109/MSP.2017.2765202

[ref10] Hamedi M. Salleh S. H. Noor A. M. (2016). Electroencephalographic motor imagery brain connectivity analysis for BCI: a review. Neural Comput. 28, 999–1041. doi: 10.1162/NECO_a_00838, PMID: 27137671

[ref11] Hartmann K. G. Schirrmeister R. T. Ball T. (2018). EEG-GAN: generative adversarial networks for electroencephalograhic (EEG) brain signals. arXiv Preprint arXiv:1806.01875. doi: 10.48550/arXiv.1806.01875

[ref12] He K.M. Zhang X.Y. Ren S.Q. Sun J. (2016). Deep residual learning for image recognition. 2016 IEEE conference on computer vision and pattern recognition (CVPR), Las Vegas, USA, 770–778.

[ref13] Herman P. Prasad G. McGinnity T. M. Coyle D. (2008). Comparative analysis of spectral approaches to feature extraction for EEG-based motor imagery classification. IEEE Trans. Neural Syst. Rehabil. Eng. 16, 317–326. doi: 10.1109/Tnsre.2008.926694, PMID: 18701380

[ref14] Ives-Deliperi V. L. Butler J. T. (2018). Relationship between EEG electrode and functional cortex in the international 10 to 20 system. J. Clin. Neurophysiol. 35, 504–509. doi: 10.1097/Wnp.0000000000000510, PMID: 30387785

[ref15] Jiang A. M. Shang J. Liu X. F. Tang Y. B. Kwan H. K. Zhu Y. P. (2020). Efficient CSP algorithm with Spatio-temporal filtering for motor imagery classification. IEEE Trans. Neural Syst. Rehabil. Eng. 28, 1006–1016. doi: 10.1109/Tnsre.2020.2979464, PMID: 32149648

[ref16] Jin J. Miao Y. Daly I. Zuo C. Hu D. Cichocki A. (2019). Correlation-based channel selection and regularized feature optimization for MI-based BCI. Neural Netw. 118, 262–270. doi: 10.1016/j.neunet.2019.07.008, PMID: 31326660

[ref17] Kim J. H. Biessmann F. Lee S. W. (2015). Decoding three-dimensional trajectory of executed and imagined arm movements from electroencephalogram signals. IEEE Trans. Neural Syst. Rehabil. Eng. 23, 867–876. doi: 10.1109/Tnsre.2014.2375879, PMID: 25474811

[ref18] Krizhevsky A. Sutskever I. Hinton G. E. (2017). ImageNet classification with deep convolutional neural networks. Commun. ACM 60, 84–90. doi: 10.1145/3065386

[ref19] Kwon O. Y. Lee M. H. Guan C. Lee S. W. (2020). Subject-independent brain-computer interfaces based on deep convolutional neural networks. IEEE Trans. Neural Netw. Learn. Syst. 31, 3839–3852. doi: 10.1109/TNNLS.2019.2946869, PMID: 31725394

[ref20] LaFleur K. Cassady K. Doud A. Shades K. Rogin E. He B. (2013). Quadcopter control in three-dimensional space using a noninvasive motor imagery-based brain-computer interface. J. Neural Eng. 10:046003. doi: 10.1088/1741-2560/10/4/046003, PMID: 23735712 PMC3839680

[ref21] Lawhern V. J. Solon A. J. Waytowich N. R. Gordon S. M. Hung C. P. Lance B. J. (2018). EEGNet: a compact convolutional neural network for EEG-based brain-computer interfaces. J. Neural Eng. 15:056013. doi: 10.1088/1741-2552/aace8c, PMID: 29932424

[ref22] LeCun Y. Kavukcuoglu K. Farabet C. (2010). Convolutional networks and applications in vision. 2010 IEEE international symposium on circuits and systems, IEEE; Paris, France: Piscataway, NJ 253–256.

[ref23] Lemm S. Blankertz B. Curio G. Muller K. R. (2005). Spatio-spectral filters for improving the classification of single trial EEG. IEEE Trans. Biomed. Eng. 52, 1541–1548. doi: 10.1109/Tbme.2005.851521, PMID: 16189967

[ref24] Liu M. Y. Huang X. Yu J. H. Wang T. C. Mallya A. (2021). Generative adversarial networks for image and video synthesis: algorithms and applications. Proc. IEEE 109, 839–862. doi: 10.1109/JPROC.2021.3049196

[ref25] Liu Y. L. Su W. B. Li Z. J. Shi G. M. Chu X. L. Kang Y. . (2019). Motor-imagery-based teleoperation of a dual-arm robot performing manipulation tasks. IEEE Trans. Cogn. Dev. Syst. 11, 414–424. doi: 10.1109/Tcds.2018.2875052

[ref26] Lotte F. Guan C. (2010). Regularizing common spatial patterns to improve BCI designs: unified theory and new algorithms. IEEE Trans. Biomed. Eng. 58, 355–362. doi: 10.1109/TBME.2010.2082539, PMID: 20889426

[ref27] Luo Y. Zhu L. Z. Wan Z. Y. Lu B. L. (2020). Data augmentation for enhancing EEG-based emotion recognition with deep generative models. J. Neural Eng. 17:056021. doi: 10.1088/1741-2552/abb580, PMID: 33052888

[ref28] Miao Y. Yin F. Zuo C. Wang X. Jin J. (2019). Improved RCSP and AdaBoost-based classification for motor-imagery BCI, 2019 IEEE international conference on computational intelligence and virtual environments for measurement systems and applications (CIVEMSA), 1–5. Piscataway, NJ IEEE.

[ref29] Nassif A. B. Shahin I. Attili I. Azzeh M. Shaalan K. (2019). Speech recognition using deep neural networks: a systematic review. IEEE Access 7, 19143–19165. doi: 10.1109/Access.2019.2896880

[ref30] Neuper C. Muller-Putz G. R. Scherer R. Pfurtscheller G. (2006). Motor imagery and EEG-based control of spelling devices and neuroprostheses. Prog Brain Res 159, 393–409. doi: 10.1016/S0079-6123(06)59025-9, PMID: 17071244

[ref31] Novi Q. Guan C. Dat T.H. Xue P. (2007). Sub-band common spatial pattern (SBCSP) for brain-computer interface. 2007 3rd international IEEE/EMBS conference on neural engineering, Kohala Coast, HI, USA: 1 and 2, 204.

[ref32] Pfurtscheller G. Brunner C. Schlogl A. da Silva F. H. L. (2006). Mu rhythm (de)synchronization and EEG single-trial classification of different motor imagery tasks. Neuroimage 31, 153–159. doi: 10.1016/j.neuroimage.2005.12.003, PMID: 16443377

[ref33] Pfurtscheller G. Neuper C. (2001). Motor imagery and direct brain-computer communication. Proc. IEEE 89, 1123–1134. doi: 10.1109/5.939829

[ref34] Ramoser H. Muller-Gerking J. Pfurtscheller G. (2000). Optimal spatial filtering of single trial EEG during imagined hand movement. IEEE Trans. Rehabil. Eng. 8, 441–446. doi: 10.1109/86.895946, PMID: 11204034

[ref35] Roy S. Dora S. McCreadie K. Prasad G. (2020). MIEEG-GAN: generating artificial motor imagery electroencephalography signals. 2020 international joint conference on neural networks (Glasgow, UK: IJCNN). 19–24 July 2020

[ref36] Saxena D. Cao J. (2021). Generative adversarial networks (GANs) challenges, solutions, and future directions. ACM Comput. Surv. 54, 1–42. doi: 10.1145/3446374

[ref37] Schirrmeister R. T. Springenberg J. T. Fiederer L. D. J. Glasstetter M. Eggensperger K. Tangermann M. . (2017). Deep learning with convolutional neural networks for EEG decoding and visualization. Hum. Brain Mapp. 38, 5391–5420. doi: 10.1002/hbm.23730, PMID: 28782865 PMC5655781

[ref38] Schmidhuber J. (2015). Deep learning in neural networks: an overview. Neural Netw. 61, 85–117. doi: 10.1016/j.neunet.2014.09.003 25462637

[ref39] Simonyan K. Zisserman A. (2014). Very deep convolutional networks for large-scale image recognition. arXiv Preprint arXiv:1409.1556. doi: 10.48550/arXiv.1409.1556

[ref40] Song Y. Yang L. Jia X. Xie L. (2021). Common spatial generative adversarial networks based EEG data augmentation for cross-subject brain-computer interface. arXiv Preprint arXiv:2102.04456. doi: 10.48550/arXiv.2102.04456

[ref41] Suk H. I. Lee S. W. (2013). A novel Bayesian framework for discriminative feature extraction in brain-computer interfaces. IEEE Trans. Pattern Anal. Mach. Intell. 35, 286–299. doi: 10.1109/Tpami.2012.69, PMID: 22431526

[ref42] Tabar Y. R. Halici U. (2017). A novel deep learning approach for classification of EEG motor imagery signals. J. Neural Eng. 14:016003. doi: 10.1088/1741-2560/14/1/016003, PMID: 27900952

[ref43] Tibshirani R. (1996). Regression shrinkage and selection via the lasso. J. R. Stat. Soc. 58, 267–288. doi: 10.1111/j.2517-6161.1996.tb02080.x

[ref44] van der Maaten L. Hinton G. (2008). Visualizing Data using t-SNE. J. Mach. Learn. Res. 9, 2579–2605.

[ref45] Voulodimos A. Doulamis N. Doulamis A. Protopapadakis E. (2018). Deep learning for computer vision: a brief review. Comput. Intell. Neurosci. 2018, 1–13. doi: 10.1155/2018/7068349, PMID: 29487619 PMC5816885

[ref46] Wang Y. Bremond F. Dantcheva A. (2021). Inmodegan: interpretable motion decomposition generative adversarial network for video generation. arXiv Preprint arXiv:2101.03049. doi: 10.48550/arXiv.2101.03049

[ref47] Xu F. Dong G. Li J. Yang Q. Wang L. Zhao Y. . (2022). Deep convolution generative adversarial network-based electroencephalogram data augmentation for post-stroke rehabilitation with motor imagery. Int. J. Neural Syst. 32:2250039. doi: 10.1142/S0129065722500393, PMID: 35881016

[ref48] Yang L. Song Y. Ma K. Xie L. (2021). Motor imagery EEG decoding method based on a discriminative feature learning strategy. IEEE Trans. Neural Syst. Rehabil. Eng. 29, 368–379. doi: 10.1109/TNSRE.2021.3051958, PMID: 33460382

[ref49] Yang X. Wang Z. H. Zhao J. Y. Yang D. (2022). FG-GAN: a fine-grained generative adversarial network for unsupervised SAR-to-optical image translation. IEEE Trans. Geosci. Remote Sens. 60, 1–11. doi: 10.1109/TGRS.2022.3165371

[ref50] Yang J. Yao S. W. Wang J. (2018). Deep fusion feature learning network for MI-EEG classification. IEEE Access 6, 79050–79059. doi: 10.1109/Access.2018.2877452

[ref51] Zhang R. Li Y. Q. Yan Y. Y. Zhang H. Wu S. Y. Yu T. Y. . (2016). Control of a wheelchair in an indoor environment based on a brain-computer Interface and automated navigation. IEEE Trans. Neural Syst. Rehabil. Eng. 24, 128–139. doi: 10.1109/Tnsre.2015.2439298, PMID: 26054072

[ref52] Zhang Q. Liu Y. (2018). Improving brain computer interface performance by data augmentation with conditional deep convolutional generative adversarial networks. arXiv Preprint arXiv:1806.07108. doi: 10.48550/arXiv.1806.07108

[ref53] Zhang D. Yao L. Chen K. Monaghan J. (2019). A convolutional recurrent attention model for subject-independent EEG signal analysis. IEEE Signal Process. Lett. 26, 715–719. doi: 10.1109/lsp.2019.2906824

[ref54] Zhang X. Yao L.N. Zhang D.L. Wang X.Z. Sheng Q.Z. Gu T. (2017). Multi-person brain activity recognition via comprehensive EEG signal analysis. Proceedings of the 14th EAI international conference on mobile and ubiquitous systems: computing, networking and services (Mobiquitous 2017), New York: VIC, Melbourne, Australia. Association for Computing Machinery 28–37.

[ref55] Zheng Z. Q. Yu Z. B. Wu Y. Zheng H. Y. Zheng B. Lee M. (2021). Generative adversarial network with multi-branch discriminator for imbalanced cross-species image-to-image translation. Neural Netw. 141, 355–371. doi: 10.1016/j.neunet.2021.04.013, PMID: 33962124

[ref56] Zou H. Hastie T. (2005). Regularization and variable selection via the elastic net. J. R. Stat. Soc. 67, 301–320. doi: 10.1111/j.1467-9868.2005.00503.x

